# Structural Discrimination in Nonprofit Hospital Community Benefit Spending

**DOI:** 10.1001/jamahealthforum.2024.5523

**Published:** 2025-02-28

**Authors:** Aaron Hedquist, David Blumenthal, Dannie Dai, Jessica Phelan, E. John Orav, Jose F. Figueroa

**Affiliations:** 1 Department of Health Policy & Management, Harvard T.H. Chan School of Public Health, Boston, Massachusetts; 2 Department of Medicine, Brigham & Women’s Hospital, Boston, Massachusetts

## Abstract

**Importance:**

Nonprofit hospitals receive substantial tax exemptions to provide a community benefit. However, little is known about the distribution of community benefit spending (CBS) across US communities with varying degrees of social vulnerability beyond the hospital’s immediate geographic area.

**Objective:**

To assess associations of CBS per capita with community-level characteristics and social determinants of health.

**Design, Setting, and Participants:**

This cross-sectional study used Internal Revenue Service Series 990 Tax Forms from 2018 to 2023, to create a dataset of CBS for nonprofit hospitals in the US. Facility-level CBS allocation to counties was based on inpatient utilization to more accurately reflect a hospital’s community. Data were analyzed from January to December 2024.

**Exposures:**

County-level race and ethnicity characteristics and socioeconomic factors, including educational attainment, proportion living below 138% of the federal poverty level (FPL), and the Social Vulnerability Index (SVI) score.

**Main Outcomes and Measures:**

The primary outcome was total CBS per capita. Generalized linear regression models with a γ log-link function were used to assess the association of CBS per capita with community-level social determinants of health characteristics.

**Results:**

A total of 2465 nonprofit hospitals across 3140 US counties were included. Allocation of CBS varied significantly across communities, with the counties in the highest quintile receiving a mean (SD) of $540 ($250) per capita compared with counties in the lowest quintile with $22 ($16) per capita. Communities in the highest quintile of CBS had a higher proportion of White residents, while communities in the lowest quintile had a higher proportion of residents who were non-Hispanic Black or Hispanic, had lower educational attainment, and were living with incomes below 138% of the FPL. For every 1% proportional increase in non-Hispanic Black or Hispanic residents in a community, there was 1.61% (95% CI, 1.38%-1.84%) and 0.88% (95% CI, 0.63%-1.14%) less CBS per capita, respectively. In addition, there was less allocation of CBS per capita among counties with a greater proportion of people with low educational attainment, greater levels of poverty, or higher SVI scores. These results were consistent before and during the COVID-19 pandemic.

**Conclusions and Relevance:**

This cross-sectional study found that nonprofit hospitals’ CBS was regressively allocated across US communities, with more socially vulnerable or racially and ethnically minoritized communities receiving less benefit than more affluent, non-Hispanic White communities, suggesting that the nonprofit tax system may be structurally discriminatory and contributing to health disparities.

## Key Points

**Question:**

How is nonprofit hospital community benefit spending allocated toward communities of varying degrees of socioeconomic vulnerability or among racially and ethnically minoritized communities?

**Findings:**

In this cross-sectional study of nonprofit hospital Internal Revenue Service tax filings from 2465 US hospitals between 2018 and 2023, communities with more social vulnerability, less educational attainment, more poverty, and more Black and Hispanic residents received less community benefit spending per capita by nonprofit hospitals, both before and during the COVID-19 pandemic.

**Meaning:**

These findings suggest that the US nonprofit hospital tax designation is structurally discriminatory against low-income and racially and ethnically minoritized communities, which may be contributing to health disparities.

## Teaser

This cross-sectional study analyzes associations of community benefit spending per capita among nonprofit hospitals in the US with community-level characteristics.

## Introduction

Nonprofit hospitals are exempt from paying income, property, and other taxes, an arrangement that costs federal, state, and local tax authorities upwards of $37.4 billion annually in foregone tax revenue.^1,2,3^ In exchange, the federal government mandates that tax-exempt hospitals must provide a community benefit, which can range from financial assistance for low-income or uninsured populations to investments in local public health programs or nonprofit organizations that address the social determinants of health (SDOH).

However, in recent years, there has been increased scrutiny of whether the nonprofit hospital designation generates enough value for communities to justify its tax exemption. Evidence also suggests that many nonprofit hospitals benefit significantly more from their tax-exempt status than they spend on community investments.^4,5^ Furthermore, in 2023, the US Government Accountability Organization expressed concern over the lack of clarity in nonprofit hospital community benefit reporting and wide variation in the activities considered to be a community benefit.^6^ In response, several states have begun to more strictly regulate reporting standards and have even imposed minimums on community benefit spending.^7,8,9,10,11,12^ Local municipalities are also increasing pressure. For example, one district in Pennsylvania successfully sued a hospital facility that led to the stripping of its local tax exemptions after the district argued the facility prioritized executive salaries over community benefits.^13^

Despite concerns surrounding the value of the nonprofit tax exemption, little is understood about where community benefit spending is spent. Given methodological challenges, the existing literature has historically defined a hospital’s community as the immediate geographic area surrounding the hospital (ie, facility zip code or county). However, hospitals located in the same zip code or county may service substantially different communities. Therefore, a broader understanding of where and how hospitals spend on community benefits would be helpful.

Community benefit regulations encourage nonprofit hospitals to redistribute profits into the communities they serve. Hospitals that disproportionately service low-income and racially and ethnically minoritized communities, however, generally have slimmer operating margins and are more likely to have a revenue shortfall.^14,15,16^ Much of this may be because safety-net hospitals are more likely to care for populations who are uninsured, which may substantially inhibit the ability of these hospitals to invest in their communities. Therefore, socially vulnerable and racially and ethnically minoritized communities may not be equitably benefiting from the current system.

To date, however, we lack empirical evidence evaluating the potential for structural discrimination in the nonprofit hospital system, which may be exacerbating health disparities. Such data may inform policymakers as they consider reforms or regulations to improve this system. Therefore, using Internal Revenue Service (IRS) tax filing data before and during the COVID-19 pandemic, we evaluated whether there are differences in the amount of community benefit spending allocated to socially vulnerable and racially and ethnically minoritized communities compared with other communities.

## Methods

### Data

This cross-sectional study was determined exempt by the Harvard T.H. Chan School of Public Health institutional review board and did not require informed consent due to the use of deidentified data in accordance with the US 45 CFR 46. The reporting of the study followed the Strengthening the Reporting of Observational Studies in Epidemiology (STROBE) reporting guideline. We used the IRS Series 990 Schedule H tax forms, which are required to be submitted by nonprofit hospitals and other tax-exempt health care organizations annually. Facilities and systems can sometimes be delayed in reporting taxes. Therefore, we extracted all IRS data released between 2020 and 2023.^17^ We divided the data into 2 cohorts for comparison. The first cohort included the latest tax return available from calendar years 2018 and 2019. The second cohort included the latest return filed after 2020 (capturing data from 2020 through 2023). A small number of hospitals (less than 1%) filed returns that straddled 2019 and 2020. We assigned these returns to their respective cohorts based on whether a majority of months of the tax period fell before or after 2020. For example, if a hospital’s tax year spanned October 2019 through September 2020, this hospital was assigned to the post-2020 cohort because it included 9 months of 2020.

Hospitals are required to report community benefit spending across 17 categories, which includes downstream medical services (eg, subsidizing Medicaid or providing uncompensated care for uninsured populations), preventive community health programming (collaborating with public health departments or financing community health initiatives), and investments in SDOH interventions of their community (Table 1). Of note, for this study, we excluded 2 measures from overall community benefit spending because these categories primarily benefit the hospital or their employees rather than the community: spending on health profession education and research. While there is no uniform definition of what is considered a community benefit, previous literature has suggested delineating more clearly between community spending that primarily benefits the hospital vs the patient or the broader community.^18^

**Table 1.** Categorization of Nonprofit Community Benefit Spending^a^

Community benefit category	Internal Revenue Service–defined subcategory
Total community benefit spending	1. Medical services and undercompensated care 2. Community health and partnerships 3. Social determinants of health
Medical services and undercompensated care	1. Financial assistance at cost 2. Medicaid shortfall 3. Other means-tested government programs 4. Subsidized health services
Community health and partnerships	1. Community health improvement services and community benefit operations 2. Cash and in-kind contributions for community benefit
Social determinants of health	1. Physical improvements and housing 2. Economic development 3. Community support 4. Environmental improvements 5. Leadership development and training for community members 6. Coalition building 7. Community health improvement advocacy 8. Workforce development 9. Other community building activities
Research and health professions education	1. Health professions education 2. Research

^a^ There is no uniform definition of what is considered a community benefit; therefore, benefits that were determined to primarily benefit the hospital based on recommendations from previous literature were excluded.

From these data, we extracted the total net spending on community benefit for each category. This measure is the total dollar amount spent on each category after taking into consideration any offsetting revenue. Previous literature has largely focused on community benefit spending as a proportion of total hospital expenses. Given our study’s focus on the health equity implications of community benefits, we analyzed dollars spent. We believe this provides a more practical understanding of resource allocation that would better highlight potential disparities.

For hospital characteristics in the 2018 to 2019 cohort, we merged data from the 2020 American Hospital Association Annual Survey Database and the 2019 Centers for Medicare & Medicaid Services (CMS) Cost Report. For hospital characteristics in the 2020 to 2023 cohort, we merged data from the Agency for Healthcare Research and Quality (AHRQ) 2022 Compendium Hospital Linkage File and the 2021 CMS Cost Report.

For community characteristics in the 2018 to 2019 cohort, we used the 2019 AHRQ County SDOH Database, which combines socioeconomic information from the American Community Survey, Area Health Resource File, and other census-derived sources.^19^ For community characteristics in the 2020 to 2023 cohort, we used the 2021 American Community Survey because more recent AHRQ data were not available at the time of study. Specifically, we included race, ethnicity, limited English proficiency, less than high school education, proportion of the population below 138% of the federal poverty level (FPL), and life expectancy. Race and ethnicity categories included non-Hispanic American Indian or Alaska Native, non-Hispanic Asian, non-Hispanic Black, Hispanic, Native Hawaiian or Pacific Islander, and non-Hispanic White. Finally, we used the 2020 Centers for Disease Control and Prevention Social Vulnerability Index (SVI), an aggregate social vulnerability measure based on socioeconomic measures.^20^

### Allocating Community Benefit Spending Across Communities

Hospitals have the option to file taxes collectively as part of a system or as a single hospital facility. For this study, single-filing hospitals were matched to a tax number using the Medicare Number to Employer Identification Number crosswalk, or when unavailable, the AHRQ Compendium of Healthcare Systems Hospital Linkage File. For hospitals that filed collectively, we first allocated IRS-defined hospital systems spending to each individual hospital following previously used methods.^4,5,21^ Specifically, we allocated a proportion of the total health system’s community benefit spending to each hospital based on the proportion of the hospital system’s uncompensated care spending attributed to each facility (based on CMS cost report uncompensated care using Worksheet S-10). We applied this proportion across each community benefit spending category to estimate the amount spent per nonprofit hospital.

To allocate spending from the facility level to the community level, we used the CMS Hospital Service Area File (which contains information on 100% of Medicare Advantage and Traditional Medicare inpatient discharges) to calculate the number of hospital discharges for each beneficiary zip code.^22^ Then, across each US zip code, we allocated a proportion of the facility’s spending equivalent to the proportion of attributable hospitalizations from that zip code. For example, if 10% of a hospital’s inpatient discharges occurred within a zip code, that zip code received a proportional 10% of that hospital’s community benefit spending. We converted zip code to county-level spending through a population-weighted crosswalk for more stable estimates and avoiding small cell size suppression requirements from CMS.

### Statistical Analysis

We conducted a cross-sectional analysis of the community benefit spending allocated to each US county based on nonprofit hospitals’ inpatient utilization using the 2020 through 2023 IRS data releases.^23^ We aggregated community benefit spending into the following 4 categories: (1) total community benefit spending, (2) medical services, (3) community health and partnerships, and (4) SDOH. We grouped counties into quintiles based on the total community benefit spending per capita. We then compared the community characteristics of counties in the top quintile, middle 3 quintiles, and the lowest quintile. Statistical significance was determined by applying a Bonferroni correction to 21 separate tests, reflecting a significance threshold of a 2-sided *P* < .0024.

Next, we constructed a series of generalized linear models with a γ log-link function to determine associations of community benefit spending with community characteristics. We chose to examine the association of community benefit spending overall (and across 3 subcategories), with each exposure variable individually because of the collinearity between community characteristics.

We conducted a series of sensitivity analysis to assess the robustness of our results. First, we weighted results by county population to better emphasize higher-population communities. Second, we performed a model that controlled for for-profit hospital and public hospital uncompensated care spending per county given that previous literature indicates that for-profit hospitals outspend local nonprofits on uncompensated care.^24^ To estimate for-profit and public spending, we allocated these facilities’ uncompensated care (as reported in the CMS Cost Report) to US counties based on inpatient utilization patterns. Third, we repeated our primary model by adding a variable for the 4 US regions. Fourth, we performed our primary model controlling for regional price parities to account for differences in price levels across states and metropolitan areas. Fifth, to more closely align with hospitals’ uncompensated care policies, we produced a sensitivity analysis that limited distribution exclusively to dual eligible Medicare and Medicaid beneficiaries or those who qualified for the Medicare Part D low-income subsidy. Sixth, we performed a multivariate model that included the proportions of Black and Hispanic individuals, percentage of those below the 138% of FPL, and percentage of those with less than a high school education as exposure variables. Finally, we conducted our primary model where community benefit spending was limited to the county where the hospital is located to compare our results with more traditional methods. Analyses were conducted using SAS version 9.4 (SAS Institute) and R version 4.4.0 (R Project for Statistical Computing) from January to December 2024.

## Results

### Nonprofit Hospital Characteristics

We identified 2465 nonprofit acute care hospitals from the 2023 IRS Series 990s electronic data release that could be linked to the AHRQ Hospital Linkage File and CMS Cost Report; this represents 94% of the nonprofit acute care hospitals in the 2022 AHRQ Hospital Linkage File and CMS Cost Report (2465 of 2634 hospitals). The characteristics differences between nonprofit hospitals with and without tax data are available in eTable 1 in Supplement 1. Of the hospitals in the sample, 1636 (66%) filed individual tax returns, while 829 (34%) filed as part of a health system. Single filers were significantly more likely to be small, rural, and nonteaching hospitals (eTable 2 in Supplement 1).

### Community Characteristics by Allocated Community Benefit Spending

Hospital community benefit spending was allocated to 3140 counties. On average, communities in the highest quintile of allocated community benefit spending received a mean (SD) of $540 ($248) per capita compared with $174 ($78) per capita in the middle 3 quintiles and $22 ($18) per capita in the lowest quintile (Table 2). Communities in the highest quintile of community benefit spending had a higher proportion of non-Hispanic White residents, while communities in the lowest quintile had a higher proportion of residents who were non-Hispanic Black or Hispanic, had lower educational attainment, and were living with incomes below 138% of the FPL. Communities with the highest quintile of community benefit spending per capita were more likely to be in the Northeast and Midwest while communities in the lowest quintile of community benefit spending were more likely located in the South (Table 2).

**Table 2.** County-Level CBS per Capita by Quintile and Community Characteristics

County characteristics	Counties by quintile of CBS per capita, mean percentage (SD) (N = 3140)			*P* value^a^
	Lowest quintile (n = 628)	Middle 3 quintiles (n = 1884)	Highest quintile (n = 628)	
Population size, mean (SD)	59 442 (149 009)	124 842 (399 818)	91 071 (232 708)	<.001
Race and ethnicity				
American Indian or Alaska Native (non-Hispanic)	2.2 (9.5)	1.6 (6.9)	2.0 (7.7)	.14
Asian (non-Hispanic)	1.2 (2.7)	1.5 (3.0)	1.3 (2.4)	.04
Black (non-Hispanic)	13.6 (17.8)	8.8 (13.9)	4.0 (8.9)	<.001
Hispanic	13.9 (18.9)	9.3 (12.5)	7.2 (11.2)	<.001
Native Hawaiian or Pacific Islander (non-Hispanic)	0.1 (0.5)	0.1 (0.4)	0.1 (0.1)	.12
White (non-Hispanic)	66.1 (21.9)	75.8 (19.4)	82.6 (17.1)	<.001
Socioeconomic factors				
Limited English proficiency	4.3 (6.1)	3.1 (4.0)	2.6 (3.7)	<.001
Education: less than high school degree	14.1 (6.9)	12.0 (5.6)	10.1 (4.7)	<.001
Less than 138% federal poverty level	24.3 (8.6)	21.5 (7.8)	20.2 (7.2)	<.001
Life expectancy, mean (SD) y	74.8 (3.4)	75.8 (3.4)	76.6 (3.3)	<.001
Social Vulnerability Index score, mean (SD)	60.4 (28.1)	49.6 (28.7)	40.8 (26.9)	<.001
Total CBS spending per capita, mean (SD), $	22 (18)	174 (78)	540 (248)	<.001
Medical services	19.8 (16.4)	158.2 (74.0)	498.6 (239.2)	<.001
Community health and partnerships	2.1 (4.2)	15.0 (19.3)	36.1 (47.3)	<.001
Social determinants of health	0.5 (0.36)	0.9 (2.6)	5.1 (10.8)	<.001
Other education and research spending	5.5 (12.6)	27.3 (30.8)	58.6 (78.8)	<.001
Region, No. (%)				
Midwest	91 (14.5)	639 (33.9)	325 (51.8)	<.001
Northeast	0	112 (5.9)	105 (16.7)	<.001
South	445 (70.9)	881 (46.8)	94 (15.0)	<.001
West	92 (14.6)	252 (13.4)	104 (16.6)	.14

Abbreviation: CBS, community benefit spending.

^a^ Unadjusted *P* values are shown. After Bonferroni correction for 21 tests, only *P* values <.0024 were considered significant.

A full breakdown of subcategory spending is available in eTable 3 in Supplement 1. Notably, a greater proportion of total community benefit spending among counties in the lowest quintile was from large hospitals, major teaching hospitals, and hospitals located in urban areas. Counties in the highest quintile had a greater proportion of community benefit spending coming from smaller hospitals and nonteaching hospitals.

### Association of Community Characteristics With Community Benefit Spending

**Figure 1.**
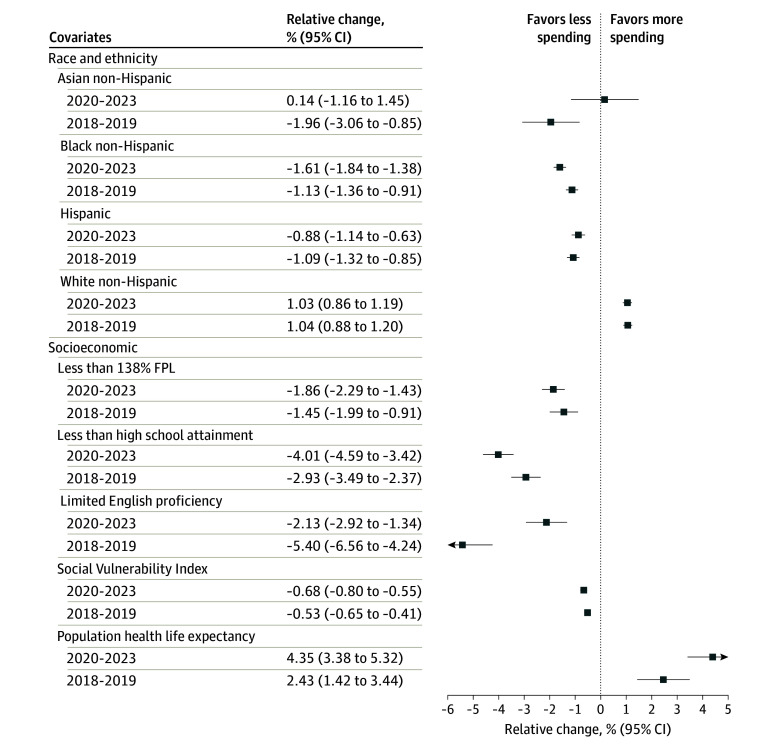
Associations of Community-Level Race and Ethnicity With Socioeconomic Characteristics and Community Benefit Spending per Capita, 2018-2023 All variables on the y-axis represent the percentage of the population in each county with that characteristic, except for the Social Vulnerability Index and life expectancy. The Social Vulnerability Index is not a percentage but does range from 0 to 100. The x-axis denotes the percentage change in net hospital spending that is associated with a 1-point increase in the Social Vulnerability Index. The x-axis denotes the percentage change in net hospital spending that is associated with a 1-year increase in life expectancy. FPL indicates federal poverty level.

In the period before and during the COVID-19 pandemic, we found that communities with more racially and ethnically minoritized populations received less community benefit spending than other communities. For every 1% increase in non-Hispanic Black residents in a community, there was 1.61% (95% CI, 1.38%-1.84%) less community spending per capita. For every 1% increase in Hispanic residents within a community, there was 0.88% (95% CI, 0.63%-1.14%) less community spending per capita. On the other hand, a 1% increase in the non-Hispanic White population within a community was associated with a 1.03% (95% CI, 0.86%-1.19%) increase in community benefit spending per capita. Similar associations were observed in analyses based on tax years before the COVID-19 pandemic in 2018 and 2019 (Figure 1).

We also found communities with higher social vulnerability were less likely to be allocated community benefit spending than other communities with less social vulnerability. For every 1% increase in the proportion of residents living below 138% of the FPL, we observed a 1.86% (95% CI, 1.43%-2.29%) decrease in community benefit spending per capita. For every 1% increase in the proportion of people who did not graduate from high school, we observed a 4.01% (95% CI, 3.42%-4.59%) decrease in community benefit spending per capita. Finally, for every 1-point increase in SVI within a community, we observed a 0.68% (95% CI, 0.55%-0.80%) decrease in community benefit spending per capita. These associations were similar in our analyses based on tax years 2018 and 2019. Finally, we observed that communities with higher life expectancy received more community benefit spending per capita. For every 1-year increase in life expectancy withing a community, we observed a 4.35% (95% CI, 3.38%-5.32%) increase in community benefit spending per capita.

**Figure 2.**
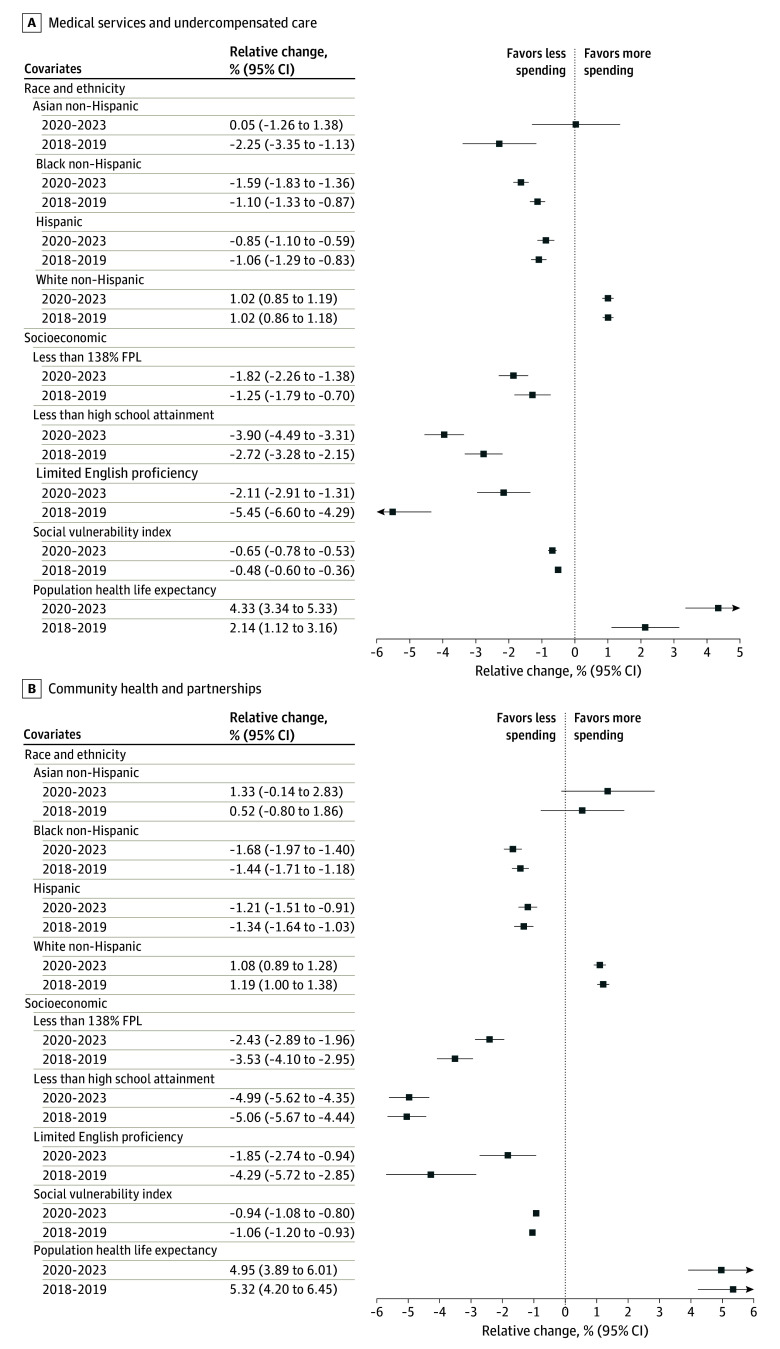
Associations of Community-Level Race and Ethnicity With Socioeconomic Characteristics and Community Benefit Spending per Capita for Medical Services and Undercompensated Care and Community Health and Partnerships, 2018-2023 FPL indicates federal poverty level.

**Figure 3.**
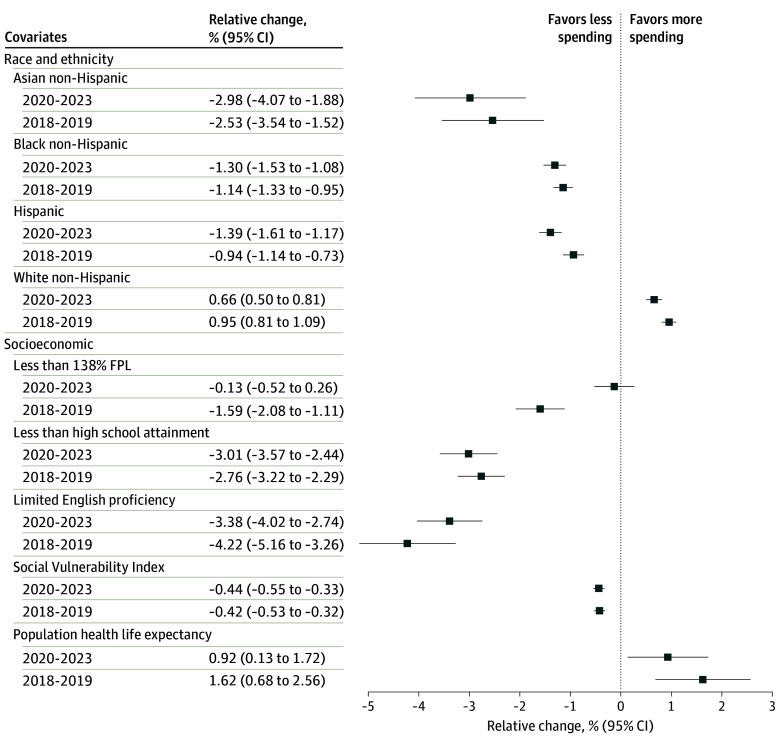
Associations of Community-Level Race and Ethnicity With Socioeconomic Characteristics and Community Benefit Spending per Capita for Social Determinants of Health, 2018-2023 FPL indicates federal poverty level.

The associations of community benefit spending with race, ethnicity, and key socioeconomic characteristics were generally consistent among the 3 subcategories of community benefit spending (Figure 2 and Figure 3). For the medical services and undercompensated care category as well as the community health and partnerships category, communities with a higher proportion of non-Hispanic Black residents, Hispanic residents, residents living below 138% of the FPL, residents who did not graduate from high school, or residents with limited English proficiency were less likely to receive community benefit spending. Similar results were observed in SDOH-related spending, with the exception that communities with more non-Hispanic Asian residents were less likely to receive SDOH-related community benefit spending. The variable of living below 138% of the FPL was also no longer significantly associated with changes in SDOH-related community benefit spending.

### Sensitivity Analysis

We conducted several sensitivity analyses. First, we included medical education and research spending in the analysis (eTable 4 in Supplement 1). These results were consistent with our main model. Second, we weighted our results by county population (eTable 4 in Supplement 1), and all results were consistent with our main model except for the associations with FPL and English proficiency, which both became nonsignificant. Third, we controlled for for-profit and public hospitals’ spending on uncompensated care and our results were consistent (eTable 4 in Supplement 1). Additionally, we limited our spending allocation to communities within 50 miles of each hospital facility, which produced consistent results with the main findings (eTable 4 in Supplement 1). For models controlling for regional price parities, we found that all results were similar to our final model except we also found that communities with more non-Hispanic Asian populations were also now significantly less likely to be allocated community benefit dollars (eTable 4 in Supplement 1).

To assess the associations within specific regions, we repeated our primary model by adding a variable for the 4 US regions; while the results became statistically nonsignificant for the socioeconomic and population health outcomes, possibly due to smaller sample sizes, the significant regressive associations of the race and ethnicity within counties with community benefit spending persisted (eTable 4 in Supplement 1). Similarly, results were consistent with our primary model after distributing spending only based on beneficiaries that were dual-eligible for Medicaid or qualified for the low-income subsidy (eTable 4 in Supplement 1). Results were also largely consistent in a multivariate model that included the proportion of non-Hispanic Black and Hispanic individuals, those with less than a high school education, and those living below the FPL as exposure variables (eTable 4 in Supplement 1). Finally, we compared our main results with the model where community benefit spending per capita was allocated only to the counties where the hospital resides. In this model, we found that many variables were no longer significant, including the Hispanic and non-Hispanic White ethnicity, the percentage below the 138% of the FPL, limited English proficiency, and SVI.

## Discussion

In this national cross-sectional study of nonprofit hospitals, we found that the allocation of community benefit spending per capita was lower among communities with more racially and ethnically minoritized or socially vulnerable people than communities with more non-Hispanic White or affluent people. Findings raise substantial concern for potential structural inequalities against racial and ethnic minority communities or those of lower socioeconomic status in the national distribution of community benefit spending.

In principle, the nonprofit hospital designation system and community benefit spending are a dividend for investment in the health of local communities. The allocation of community benefit spending per capita unfortunately appears to be regressive, with communities that may need assistance the most receiving less support. Our study focused on examining absolute spending per capita rather than the proportion of a hospital’s operating expenses because this ultimately is what determines the extent to which people may benefit from social interventions, community partnerships, and programs. Even in the wake of the COVID-19 pandemic, which exposed and worsened health inequities that exist in our country, the associated decreases in community benefit spending among more socially vulnerable or racially and ethnically minoritized people persisted and maybe even worsened when compared with data prior to the COVID-19 pandemic. In addition, it is substantially concerning that communities with lower life expectancy at baseline are receiving less of a community benefit than those with much higher life expectancy.

Our results, therefore, raise concern that the current system may be a contributor to existing racial and ethnic health disparities rather than an alleviator. It is well documented that structural discrimination is embedded throughout the health care system in the US, with racially and ethnically minoritized populations having lower quality care, limited access to insurance, and poorer financial support from servicing facilities.^25^ Prior work has also found that allocation of the Medicare and Medicaid Disproportionate Share Hospital payment programs, which allocated $24 billion to hospitals annually to subsidize care for low-income patients, is structurally discriminatory against low-income racial and ethnic minority communities. Our work suggests that the same concern exists in the nonprofit hospital tax system, where we have a system that is seemingly neutral in nature but is unintentionally structurally discriminatory because it disproportionately negatively impacts certain socially vulnerable and racially and ethnically minoritized groups.

Moving forward, it will be important for policymakers and clinical leaders to consider policy changes which would better allocate investments into more socially vulnerable communities. Hospitals and health systems have an important role in addressing racial disparities, alleviating the burden of medical debt among low-income individuals, and improving the health of racially and ethnically minoritized populations.^26^ Community benefit programming is an opportunity for nonprofit hospitals to do just this by providing care at lower costs for those unable to pay, building community networks for health, and filling the gap in SDOH coverage when local safety nets are unable to provide for communities.

### Strengths and Limitations

This study builds upon previous literature on the impact and allocation of community benefit programming by shifting the definition of a hospital’s community away from narrow geographic settings^18,21,27,28,29,30^ to instead a novel approach of allocating community benefit spending based on the location of the treated hospitalized population of a given hospital. For example, an earlier study^22^ that examined hospital community benefit spending and local socioeconomic characteristics limited spending to the immediate zip code. However, a hospital’s community is largely not bound by county or zip code borders. In fact, 98.7% and 43.1% of health care referral regions cross county and state lines, respectively.^31^ Earlier work often cites the absence of clear community definition as a limitation in analysis.^21,22^ In this context, our study offers a more novel method to help address some of these limitations. Instead of applying a uniform geographic limit to each hospital’s spending radius, we distributed dollars based on actual patient utilization from each community. We believe this emphasis on patient-centered communities may more accurately reflect how hospitals distribute community funds, especially because the majority of spending is on medical services.

However, several limitations to our study should be taken into consideration. First, the underlying tax filing data are subject to reporting bias. The IRS provides only limited guidance on the definition of community benefit categories; this leaves the interpretation of what and how to report to individual facilities and hospital systems.^18,32^ Therefore, nonprofit hospitals might misrepresent their spending or revenue. Similarly, spending on SDOH was extracted from Part II of the IRS Schedule H, which has different reporting guidelines that may introduce bias. In addition, it is important to note that compared with the amount in the medical spending category, spending on SDOH is quite small in absolute terms. Yet the fact that spending on addressing social needs of populations appears to be regressively allocated across US communities is still of major concern, and further work is needed to understand the practical significance of this inequitable distribution.

Furthermore, our method of allocating community benefit spending based only on Medicare inpatient utilization is a limitation. It is important to note, however, that our method for modeling local area–level spending estimates is supported by prior validated work by the Dartmouth Health Atlas showing that medical-related travel patterns between the general population and Medicare-aged populations were similar.^33^ In addition, our study is an observational cross-sectional analysis that limits our ability to establish causality between community-level variables and community benefit spending. However, the intent of our study was to assess whether community benefit spending was being differentially allocated toward communities who may be in more need and stand to benefit from relief. Additionally, this study does not allow us to determine how best policymakers should consider reforms or regulations for the current nonprofit tax system. Further research and analysis are needed to understand the implications of major reforms to ensure a more equitable system. Our findings do, however, suggest that the current system is having consequences that merit a careful review to ensure that the tax exemptions for nonprofit hospitals result in a more equitable distribution of the resulting tax benefits.

## Conclusions

In this cross-sectional study using US national tax data, we found that more socially vulnerable and racially and ethnically minoritized communities are receiving less community benefit spending by nonprofit hospitals, raising concern that the current system is regressive and potentially structurally discriminatory. Moving forward, policymakers should consider reforms that address a more equitable allocation of resources to communities that may need it most to help alleviate systemwide health inequities.

## Notes

Supplement 1. **eTable 1.** Characteristics of Hospitals With Linked Tax Records vs Hospitals With No Tax Records **eTable 2.** Characteristics of Single-Filing Hospitals vs Multifiling Hospitals **eTable 3.** County-Level Subcategory Community Benefit Spending by Quintile **eTable 4.** Sensitivity Analyses Using Models That Account for County-Level For-Profit Spending and Public Spending, Population-Level Weighted Results, US Regions, and Regional Price Parities, With Attribution Limited to Only Beneficiaries Dually Eligible for Medicaid or Low-Income Subsidy

Supplement 2.
Data Sharing Statement
