# Supplementary material for: Structural Discrimination in Nonprofit Hospital Community Benefit Spending
Source: JAMA Health Forum. 2025 Feb 28;6(2):e245523. doi: 10.1001/jamahealthforum.2024.5523 (PMC11871545; doi:10.1001/jamahealthforum.2024.5523)
Supplement: Supplement 1. — eTable 1. Characteristics of Hospitals With Linked Tax Records vs Hospitals With No Tax Records eTable 2. Characteristics of Single-Filing Hospitals vs Multifiling Hospitals eTable 3. County-Level Subcategory Community Benefit Spending by Quintile eTable 4. Sensitivity Analyses Using Models That Account for County-Level For-Profit Spending and Public Spending, Population-Level Weighted Results, US Regions, and Regional Price Parities, With Attribution Limited to Only Beneficiaries Dually Eligible for Medicaid or Low-Income Subsidy [file jamahealthforum-e245523-s001.pdf]

Supplemental Online Content

Hedquist A, Blumenthal D, Dai D, Phelan J, Orav EJ, Figueroa JF. Structural discrimination in nonprofit hospital community benefit spending. *JAMA Health Forum*. Published online February 28, 2025. doi:10.1001/jamahealthforum.2024.552

eTable 1. Characteristics of Hospitals With Linked Tax Records vs Hospitals With No Tax Records
eTable 2. Characteristics of Single-Filing Hospitals vs Multifiling Hospitals
eTable 3. County-Level Subcategory Community Benefit Spending by Quintile
eTable 4. Sensitivity Analyses Using Models That Account for County-Level For-Profit Spending and Public Spending, Population-Level Weighted Results, US Regions, and Regional Price Parities With Attribution Limited to Only Beneficiaries Dually Eligible for Medicaid or Low-Income Subsidy

This supplemental material has been provided by the authors to give readers additional information about their work.

© 2025 Hedquist A et al. *JAMA Health Forum*.

eTable 1. Characteristics of Hospitals With Linked Tax Records vs Hospitals With No Tax Records

	Hospitals with no Linkable Tax	Hospitals with Linkable Tax Records	P-value
**No. of Hospitals**	169	2,465	
**Number of Beds, Mean (SD)**	76.6 (156.1)	173.8 (218.0)	<0.001
**Hospital Size, N (%)**			
Small	117 (69.2%)	1,225 (49.7%)	<0.001
Medium	13 (7.7%)	970 (39.4%)	
Large	7 (4.1%)	270 (11.0%)	
**Region, N (%)**			
Midwest	51 (30.2%)	894 (36.3%)	<0.001
Northeast	13 (7.7%)	430 (17.4%)	
South	64 (37.9%)	682 (27.7%)	
West	41 (24.3%)	459 (18.6%)	
**Urbanicity, N (%)**			
Urban/Suburban	144 (85.2%)	2,320 (94.1%)	<0.001
Rural	25 (14.8%)	145 (5.9%)	
**Teaching Status, N (%)**			
Major Teaching	9 (5.3%)	261 (10.6%)	<0.001
Minor Teaching	8 (4.7%)	609 (24.7%)	
Not Teaching	152 (89.9%)	1,595 (64.7%)	
**Safety Net Status, N (%)**			
Lower 3 Quartiles DSH	124 (73.4%)	2,186 (88.7%)	0.15
Top Quartile DSH	13 (7.7%)	279 (11.3%)	
**Critical Access Hospitals, N (%)**	88 (52.1%)	668 (27.1%)	<0.001
**Total Admissions, Mean (SD)**	2,958.0 (7275.2)	8,044.4 (10811.8)	<0.001
**Medicare Admissions, Mean (SD)**	1,098.0 (2634.9)	3,577.8 (4556.6)	<0.001

Note: Cells that contain count values list the count and the percentage of the total count in the column in parenthesis. Cells that contain mean values list the standard deviation in parenthesis. Rural-Urban Continuum Codes (RUCC) were used to classify counties as Urban/Suburban (RUCC codes 1-7) or Rural (RUCC codes 8-9).

© 2025 Hedquist A et al. *JAMA Health Forum*.

eTable 2. Characteristics of Single-Filing Hospitals vs Multifiling Hospitals

	Facilities Belonging to Multi-Filers	Facilities Belonging to Single-Filers	P-value
**No. of Hospitals (N)**	829	1636	
**No. of Beds, Mean (SD)**	202.5 (229.7)	159.3 (210.4)	<0.001
**Hospital Size, N (%)**			
Small	334 (40.3%)	891 (54.5%)	<0.001
Medium	381 (46.0%)	589 (36.0%)	
Large	114 (13.8%)	156 (9.5%)	
**Region, N (%)**			
Midwest	274 (33.1%)	620 (37.9%)	<0.001
Northeast	91 (11.0%)	339 (20.7%)	
South	223 (26.9%)	459 (28.1%)	
West	241 (29.1%)	218 (13.3%)	
**Urban/Rural, N (%)**			
Metro	668 (80.6%)	1066 (65.2%)	<0.001
Non-Metro	161 (19.4%)	570 (34.8%)	
Urban/Suburban	800 (96.5%)	1520 (92.9%)	<0.001
Rural	29 (3.5%)	116 (7.1%)	
**Teaching Status, N (%)**			
Major Teaching	101 (12.2%)	160 (9.8%)	<0.001
Minor Teaching	265 (32.0%)	344 (21.0%)	
Not Teaching	463 (55.9%)	1132 (69.2%)	
**Safety Net Status, N (%)**			
Lower Three Quartiles of DSH	715 (86.2%)	1471 (89.9%)	0.01
Top Quartile of DSH	114 (13.8%)	165 (10.1%)	
**Critical Access Hospitals, N (%)**	148 (17.9%)	520 (31.8%)	<0.001
**Total Admissions, Mean (SD)**	9693.2 (11766.2)	7209.0 (10196.9)	<0.001
**Medicare Admissions, Mean (SD)**	4164.6 (4697.9)	3280.5 (4455.4)	<0.001

© 2025 Hedquist A et al. *JAMA Health Forum*.

eTable 3. County-Level Subcategory Community Benefit Spending by Quintile

	Overall (n = 3140)	Counties in Lowest Quintile of CBS per capita (n = 628)	Counties in the Middle Three Quintiles of CBS per capita (n = 1884)	Counties in the Highest Quintile of CBS per capita (n = 628)
	Mean (SD)	Mean (SD)	Mean (SD)	Mean (SD)
**Net CBS Spending (per Capita)**	$217.03 (213.32)	$22.16 (18.26)	$174.39 (77.73)	$539.82 (247.93)
**Medical Services & Undercompensated Care**	$198.60 (200.35)	$19.76 (16.44)	$158.24 (74.00)	$498.55 (239.15)
Financial Assistance of Cost	$42.08 (44.94)	$8.17 (10.53)	$43.12 (35.98)	$72.86 (63.32)
Medicaid Shortfall	$101.99 (120.34)	$8.33 (10.62)	$82.33 (63.75)	$254.65 (163.99)
Other Means-Tested Government Programs	$2.69 (14.78)	$0.35 (1.43)	$2.00 (8.16)	$7.11 (29.42)
Subsidized Health Services	$51.85 (111.88)	$2.93 (5.34)	$30.79 (41.48)	$163.94 (202.86)
**Community Health and Partnerships**	$16.64 (28.18)	$2.12 (4.21)	$15.00 (19.37)	$36.05 (47.28)
Community Health Improvement Services	$8.77 (15.86)	$1.11 (1.98)	$7.51 (9.86)	$20.22 (27.72)
Cash Contribution	$7.91 (20.45)	$1.15 (3.20)	$7.52 (15.37)	$15.84 (35.56)
**Social Determinants of Health**	$1.58 (5.51)	$0.05 (0.36)	$0.94 (2.55)	$5.05 (10.80)
Physical Improvements and Housing	$0.07 (0.53)	$0.00 (0.01)	$0.03 (0.12)	$0.27 (1.14)
Economic Development	$0.07 (0.39)	$0.01 (0.05)	$0.05 (0.24)	$0.20 (0.76)
Community Support	$0.61 (2.96)	$0.06 (0.26)	$0.32 (0.93)	$2.01 (6.23)
Environmental Improvements	$0.02 (0.18)	$0.00 (0.01)	$0.02 (0.17)	$0.05 (0.29)
Leadership Development and Training for Community Members	$0.02 (0.16)	$0.00 (0.01)	$0.01 (0.08)	$0.06 (0.34)
Coalition Building	$0.05 (0.38)	$0.00 (0.04)	$0.04 (0.25)	$0.14 (0.71)
Community Health Improvement Advocacy	$0.30 (2.11)	$0.01 (0.05)	$0.19 (1.09)	$0.90 (4.27)
Workforce Development	$0.45 (1.97)	$0.03 (0.17)	$0.32 (1.16)	$1.26 (3.80)
Other Community Building Activities	$0.16 (1.33)	$0.01 (0.07)	$0.16 (1.00)	$0.33 (2.41)
**Other Community Benefit**	$29.19 (46.17)	$5.48 (12.59)	$27.29 (30.84)	$58.58 (78.85)
Research	$4.87 (18.43)	$0.49 (1.60)	$3.56 (8.77)	$13.15 (37.07)
Health Professions Education	$24.35 (32.68)	$5.09 (11.93)	$23.74 (25.74)	$45.43 (48.95)
**Source of County-Level Proportion of Community Benefit Spending by Hospital Characteristics**				
**Hospital Size**	25.2% (30.1%)	15.2% (27.6%)	22.8% (27.6%)	40.6% (33.5%)
Small	41.5% (35.0%)	40.7% (39.4%)	43.9% (34.1%)	35.2% (32.9%)
Medium	33.3% (33.2%)	44.1% (39.1%)	33.3% (32.2%)	24.2% (27.8%)
Large				
**Teaching Status**	17.7% (27.1%)	19.6% (33.0%)	18.1% (26.2%)	14.9% (24.2%)
Major	39.9% (34.4%)	44.7% (40.2%)	41.1% (33.4%)	32.4% (30.5%)
Minor	42.4% (35.1%)	35.7% (38.3%)	40.8% (34.0%)	52.7% (33.4%)
Non-teaching				
**Urbanicity**				
Urban/Suburban	97.3% (12.2%)	99.3% (6.4%)	97.8% (10.5%)	94.0% (18.6%)
Rural	2.7% (12.2%)	0.7% (6.4%)	2.2% (10.5%)	6.0% (18.6%)

Note: P-values across quintiles for each variable were <0.001

© 2025 Hedquist A et al. *JAMA Health Forum.*

eTable 4. Sensitivity Analyses Using Models That Account for County-Level For-Profit Spending and Public Spending, Population-Level Weighted Results, US Regions, and Regional Price Parities, With Attribution Limited to Only Beneficiaries Dually Eligible for Medicaid or Low-Income Subsidy

Covariates	% Change in CBS Spending	95% CI	P-value
**A. Models Including Research and Education Spending**			
**Race and Ethnicity (per 1 percentage-point increase)**			
Asian Non-Hispanic	0.74%	-0.59%, 2.10%	0.277
Black Non-Hispanic	-1.41%	-1.64%, -1.18%	<.0001
Hispanic	-0.97%	-1.22%, -0.72%	<.0001
White Non-Hispanic	0.99%	0.82%, 1.16%	<.0001
**Socioeconomic Factors (per 1 percentage-point increase)**			
Below 138% FPL	-1.92%	-2.35%, -1.48%	<.0001
Less Than High School Attainment	-3.99%	-4.57%, -3.40%	<.0001
Limited English Households	-1.95%	-2.74%, -1.16%	<.0001
Social Vulnerability Index	-0.66%	-0.78%, -0.53%	<.0001
**Population Health at Baseline (per 1yr-increase in life expectancy in county)**			
Life Expectancy	4.36%	3.39%, 5.33%	<.0001
**B. Models Adjusting For County-Level For-Profit and Public Hospital Spending**			
**Race and Ethnicity (per 1 percentage-point increase)**			
Asian Non-Hispanic	0.23%	-1.06%, 1.53%	0.729
Black Non-Hispanic	-1.59%	-1.82%, -1.36%	<0.001
Hispanic	-0.78%	-1.04%, -0.53%	<0.001
White Non-Hispanic	0.97%	0.80%, 1.14%	<0.001
**Socioeconomic Factors (per 1 percentage-point increase)**			
Below 138% FPL	-1.82%	-2.25%, -1.38%	<0.001
Less Than High School Attainment	-3.81%	-4.39%, -3.22%	<0.001
Limited English Households	-1.78%	-2.58%, -0.99%	<0.001
Social Vulnerability Index	-0.65%	-0.77%, -0.52%	<0.001
**Population Health at Baseline (per 1yr-increase in life expectancy in county)**			
Life Expectancy	4.32%	3.35%, 5.29%	<0.001
**C. Models that Use County-Level Population Weighted Results**			
**Race and Ethnicity (per 1 percentage-point increase)**			
Asian Non-Hispanic	0.65%	0.25%, 1.06%	0.001
Black Non-Hispanic	-0.53%	-0.74%, 0.32%	<0.001
Hispanic	-0.56%	-0.72%, -0.39%	<0.001
White Non-Hispanic	0.36%	0.25%, 0.48%	<0.001
**Socioeconomic Factors (per 1 percentage-point increase)**			
Below 138% FPL	-0.30%	-0.72%, 0.12%	0.161
Less Than High School Attainment	-0.93%	-1.45%, 0.40%	<0.001

© 2025 Hedquist A et al. *JAMA Health Forum*.

Limited English Households	0.35%	0.00%, 0.71%	0.053
Social Vulnerability Index	-0.11%	-0.21%, 0.00%	0.041
**Population Health at Baseline (per 1yr-increase in life expectancy in county)**			
Life Expectancy	3.26%	2.33%, 4.19%	<0.001
**D. Models Adjusting For US Regions**			
**Race and Ethnicity (per 1 percentage-point increase)**			
Asian Non-Hispanic	-0.98%	-2.17%, 0.23%	0.111
Black Non-Hispanic	-0.40%	-0.67%, -0.13%	0.003
Hispanic	-0.50%	-0.74%, -0.25%	<0.001
White Non-Hispanic	0.44%	0.26%, 0.62%	<0.001
**Socioeconomic Factors (per 1 percentage-point increase)**			
Below 138% FPL	0.10%	-0.38%, 0.57%	0.688
Less Than High School Attainment	-0.70%	-1.40%, 0.01%	0.054
Limited English Households	-2.09%	-2.88%, -1.30%	<0.001
Social Vulnerability Index	-0.06%	-0.19%, 0.08%	0.42
**Population Health at Baseline (per 1yr-increase in life expectancy in county)**			
Life Expectancy	-0.32%	-1.38%, 0.76%	0.564
**E. Models Where Allocation is Limited to Communities within 50-miles of each Hospital**			
**Race and Ethnicity (per 1 percentage-point increase)**			
Asian Non-Hispanic	-0.03%	-1.30%, 1.26%	0.963
Black Non-Hispanic	-1.44%	-1.67%, -1.21%	<0.001
Hispanic	-0.71%	-0.97%, -0.46%	<0.001
White Non-Hispanic	0.90%	0.73%, 1.07%	<0.001
**Socioeconomic Factors (per 1 percentage-point increase)**			
Below 138% FPL	-1.59%	-2.03%, -1.15%	<0.001
Less Than High School Attainment	-3.40%	-4.00%, -2.80%	<0.001
Limited English Households	-1.79%	-2.57%, -1.01%	<0.001
Social Vulnerability Index	-0.55%	-0.68%, -0.43%	<0.001
**Population Health at Baseline (per 1yr-increase in life expectancy in county)**			
Life Expectancy	3.61%	2.63%, 4.59%	<0.001
**F. Models Attributed by Beneficiaries Eligible for Medicaid or Low-Income Subsidy Only**			
**Race and Ethnicity (per 1 percentage-point increase)**			
Asian Non-Hispanic	-0.71%	-1.75%, 0.34%	0.183
Black Non-Hispanic	-1.19%	-1.38%, -1.00%	<0.001
Hispanic	-0.64%	-0.85%, -0.43%	<0.001
White Non-Hispanic	0.61%	0.47%, 0.75%	<0.001
**Socioeconomic Factors (per 1 percentage-point increase)**			
Below 138% FPL	-0.16%	-0.54%, 0.23%	0.423
Less Than High School Attainment	-2.42%	-2.94%, -1.90%	<0.001
Limited English Households	-1.77%	-2.44%, -1.11%	<0.001

© 2025 Hedquist A et al. *JAMA Health Forum*.

Social Vulnerability Index	-0.31%	-0.41%, -0.20%	<0.001
**Population Health at Baseline (per 1yr-increase in life expectancy in county)**			
Life Expectancy	0.99%	0.18%, 1.82%	0.017
**G. Models with Regional Price Parities (RPP) Standardized Spending Per Capita**			
**Race and Ethnicity (per 1 percentage-point increase)**			
Asian Non-Hispanic	-1.34%	-2.56%, -0.10%	0.035
Black Non-Hispanic	-1.58%	-1.81%, -1.35%	<0.001
Hispanic	-0.99%	-1.23%, -0.74%	<0.001
White Non-Hispanic	1.09%	0.93%, 1.26%	<0.001
**Socioeconomic Factors (per 1 percentage-point increase)**			
Below 138% FPL	-1.53%	-1.97%, -1.09%	<0.001
Less Than High School Attainment	-3.74%	-4.33%, -3.14%	<0.001
Limited English Households	-2.84%	-3.61%, -2.07%	<0.001
Social Vulnerability Index	-0.64%	-0.76%, -0.51%	<0.001
**Population Health at Baseline (per 1yr-increase in life expectancy in county)**			
Life Expectancy	3.51%	2.54%, 4.50%	<0.001
**H. Multivariate Model Including % Black, % Hispanic, % below FPL, and % < High School Education as Predictors**			
**Race and Ethnicity (per 1 percentage-point increase)**			
Black Non-Hispanic	-1.58%	-1.81%, -1.35%	<0.001
Hispanic	-0.99%	-1.23%, -0.74%	<0.001
**Socioeconomic Factors (per 1 percentage-point increase)**			
Below 138% FPL	-1.53%	-1.97%, -1.09%	<0.001
Less Than High School Attainment	-3.74%	-4.33%, -3.14%	<0.001
**I. Models Limiting Community Benefit Spending Per Capita to Counties Where Hospital is Located**			
**Race and Ethnicity (per 1 percentage-point increase)**			
Asian Non-Hispanic	1.11%	-0.45%, 2.69%	0.163
Black Non-Hispanic	-0.48%	-0.88%, -0.08%	0.019
Hispanic	-0.04%	-0.46%, 0.39%	0.064
White Non-Hispanic	0.15%	-0.11%, 0.42%	0.252
**Socioeconomic Factors (per 1 percentage-point increase)**			
Below 138% FPL	-0.21%	-0.98%, 0.55%	0.584
Less Than High School Attainment	-2.10%	-3.04%, -1.16%	<0.001
Limited English Households	0.30%	-0.87%, 1.48%	0.619
Social Vulnerability Index	-0.07%	-0.26%, 0.11%	0.437
**Population Health at Baseline (per 1yr-increase in life expectancy in county)**			
Life Expectancy	2.81	1.19, 4.45	<0.001

© 2025 Hedquist A et al. *JAMA Health Forum*.
