# Supplementary material for: Structural Discrimination in Nonprofit Hospital Community Benefit Spending
Source: JAMA Health Forum. 2025 Feb 28;6(2):e245523. doi: 10.1001/jamahealthforum.2024.5523 (PMC11871545; doi:10.1001/jamahealthforum.2024.5523)
Supplement: Supplement 2. — Data Sharing Statement [file jamahealthforum-e245523-s002.pdf]

# Data Sharing Statement

Hedquist. Structural Discrimination in Nonprofit Hospital Community Benefit Spending. *JAMA Health Forum*. Published February 28, 2025. doi:10.1001/jamahealthforum.2024.5523

**Data**
**Data available:** No

**Additional Information**
**Explanation for why data not available:** Some of our data in this study is protected research identifiable data from the Centers for Medicare and Medicaid Services. Individuals interested in this research can access the same data by applying for their own data use agreement.
